# Locomotor training through a novel robotic platform for gait rehabilitation in pediatric population: short report

**DOI:** 10.1186/s12984-016-0206-x

**Published:** 2016-11-14

**Authors:** C. Bayón, S. Lerma, O. Ramírez, J.I. Serrano, M.D. Del Castillo, R. Raya, J.M. Belda-Lois, I. Martínez, E. Rocon

**Affiliations:** 1 Neural and Cognitive Engineering group, Centro de Automática y Robótica, Consejo Superior de Investigaciones Científicas, Ctra Campo Real km 0.2, Arganda del Rey, Madrid, 28500 Spain; 2 Hospital Infantil Universitario Niño Jesús, Madrid, Spain; 3 Instituto de Biomecánica de Valencia, Valencia, Spain; 4 Postgraduate Program, Universidade Federal do Espírito Santo, Vitória, Brazil

**Keywords:** Cerebral palsy, Rehabilitation robotic, Gait, Posture, Exoskeleton device, Spastic diplegia, Case report

## Abstract

**Background:**

Cerebral Palsy (CP) is a disorder of posture and movement due to a defect in the immature brain. The use of robotic devices as alternative treatment to improve the gait function in patients with CP has increased. Nevertheless, current gait trainers are focused on controlling complete joint trajectories, avoiding postural control and the adaptation of the therapy to a specific patient. This paper presents the applicability of a new robotic platform called CPWalker in children with spastic diplegia.

**Findings:**

CPWalker consists of a smart walker with body weight and autonomous locomotion support and an exoskeleton for joint motion support. Likewise, CPWalker enables strategies to improve postural control during walking. The integrated robotic platform provides means for testing novel gait rehabilitation therapies in subjects with CP and similar motor disorders. Patient-tailored therapies were programmed in the device for its evaluation in three children with spastic diplegia for 5 weeks.

After ten sessions of personalized training with CPWalker, the children improved the mean velocity (51.94 ± 41.97 %), cadence (29.19 ± 33.36 %) and step length (26.49 ± 19.58 %) in each leg. Post-3D gait assessments provided kinematic outcomes closer to normal values than Pre-3D assessments.

**Conclusions:**

The results show the potential of the novel robotic platform to serve as a rehabilitation tool. The autonomous locomotion and impedance control enhanced the children’s participation during therapies. Moreover, participants’ postural control was substantially improved, which indicates the usefulness of the approach based on promoting the patient’s trunk control while the locomotion therapy is executed. Although results are promising, further studies with bigger sample size are required.

**Electronic supplementary material:**

The online version of this article (doi:10.1186/s12984-016-0206-x) contains supplementary material, which is available to authorized users.

## Background

One of the most important consequences of Cerebral Palsy (CP) in children is mobility impairment, characterized by reduced speed and endurance or shortened step length during gait [1]. In last decades, robot-based therapy has complemented conventional strategies in CP gait rehabilitation [2, 3]. Several rehabilitation devices have been recently proposed with this aim [4, 5]. The majority of these platforms (e.g. Lokomat [4], GT-1 [5]) includes the approach of Partial Body-Weight Support (PBWS) and guided and repetitive movement. However, therapies carried out with common available devices are similar for all subjects, despite the cognitive and physical differences among them make important to tailor the therapy to the specific patient’s needs. Taking into account that maintaining a proper posture during walking is a relevant aspect in the case of children with CP [6, 7], new strategies are needed to improve postural control while over-ground movement is allowed.

In order to address these limitations, a novel robotic platform (CPWalker [8]) has been designed. It can implement new types of therapies in children with CP. The shift that CPWalker introduces on the treatments is supported by three main pillars: a) the option of free and over-ground movement (not restricted to treadmill) in rehabilitation environment; b) the improvement of postural control of head and trunk using a biofeedback strategy; and c) the use of “Assist As Needed” (AAN) strategies in specific and selected subtasks of walking.

## Methods

### Patients

Three pediatric patients with spastic diplegia (one female), two suffering from spastic CP and one from Hereditary Spastic Paraparesis (HSP), were recruited to participate in this study (Table 1). The inclusion criteria for the patient’s recruitment was: a) capable of understanding the proposed exercises; b) aged 11 to 18 years; c) maximum weight 75 kg; d) children with no deformations that could prevent the use of the exoskeleton; e) Gross Motor Function Classification System (GMFCS) levels I to III; f) able to signal pain or discomfort. The exclusion criteria of this study was defined as: a) unhealed skin lesions in the lower limbs; b) aggressive or self-harming behaviors; c) severe cognitive impairment.

**Table 1:** Description of the patients

Patient	Disease	GMFCS	Age	Weight [kg]
Patient 1	Spastic diplegia-CP	III	14	32
Patient 2	Spastic diplegia-CP	II	12	40
Patient 3	Spastic diplegia-HSP	-	13	43

The clinical trial was carried out at “Hospital Infantil Universitario Niño Jesús”. The Local Ethical Committee of this hospital gave approval to the study, and warranted its accordance with the Declaration of Helsinki. All patients and families were informed beforehand, and provided consent through parents to participate.

### Experimental apparatus

**Fig. 1:**
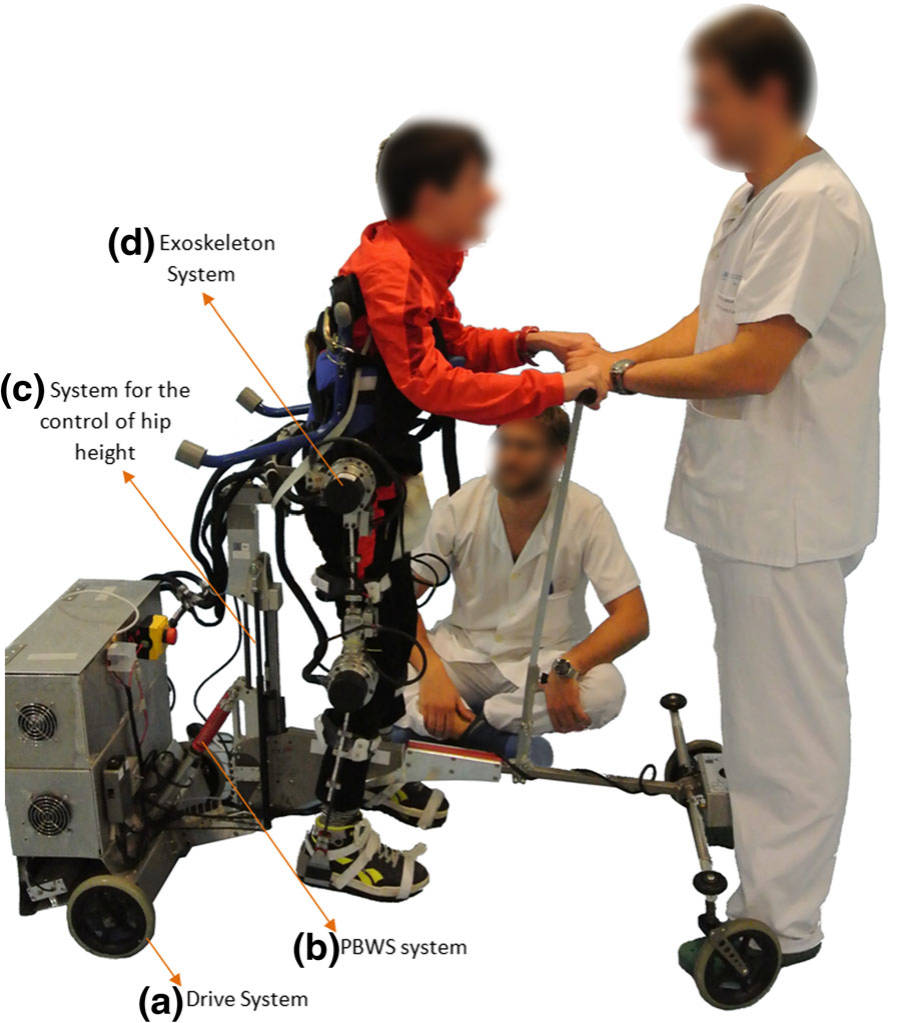
Final view of the CPWalker Platform. *a* Drive system which provides free movement of the platform; *b* PBWS system that allow the possibility of choosing the discharge of weight that support the child’s lower limbs; *c* System for the control of hip height in order to adapt the platform to different anthropometric measures; *d* Exoskeleton system with actuated joints in hip and knee

The platform used in this study to help the users to recover the gait function is the robotic trainer CPWalker (Fig. 1) described in detail in [8]. CPWalker incorporates actuators in both the walker and the exoskeleton, to constitute an active rehabilitation robotic platform. The included systems, showed in Fig. 1, are: a) a drive system to provide the translation movement required for an ambulation treatment in real rehabilitation environments instead of treadmill training; b) PBWS system to unload the child from the ground with the therapeutic benefits of improving balance and other characteristics such as symmetry or stride length [9, 10]; c) a system for the control of hip height, to adapt the robotic platform to different anthropometric measures; and d) a six Degrees Of Freedom (6-DOF) adjustable exoskeleton system for providing guided movements in sagittal plane. A video showing the experimentations with patients is available in the Additional file 1.

### Therapy

The training with CPWalker was performed for 5 weeks, 2 days per week (10 training sessions), with the exercise time for each day set at 60 min, including 10 min of setup time. The exercises consisted of walking with the robotic device through routes in flat and straight lines into the hospital facilities.

The therapies were individually adapted for each patient aiming at enhancing the most affected region in each case (according to the results of gait assessments done before to start with robot-based therapies). Specifically, the treatment defined for Patient 1 and Patient 3 attempted to improve the postural control of the trunk during walking. As a result, a stiffer impedance control of the lower limbs joints was set to assist the users’ movement while they were more focused on posture control using a biofeedback strategy. On the other hand, with Patient 2 the main purpose was to improve the range of motion (ROM) of the hip joint, primarily the extension movement. In order to address this rehabilitation, a less stiff impedance control of this joint was adjusted to intensify Patient 2’s collaboration in reaching the maximum extension of the hip. Given that this child had rigid Ankle-Foot Orthoses (AFO), the ankle joints were fixed to 90°. With no actuation on ankles, the propulsion on the ground was not high enough, so we imposed position control to knee joints in order to achieve a proper knee flexion. For this patient the biofeedback strategy for postural control was set with a bigger tolerance, which enabled the patient to be more focused on improving the hip movement.

**Fig. 2:**
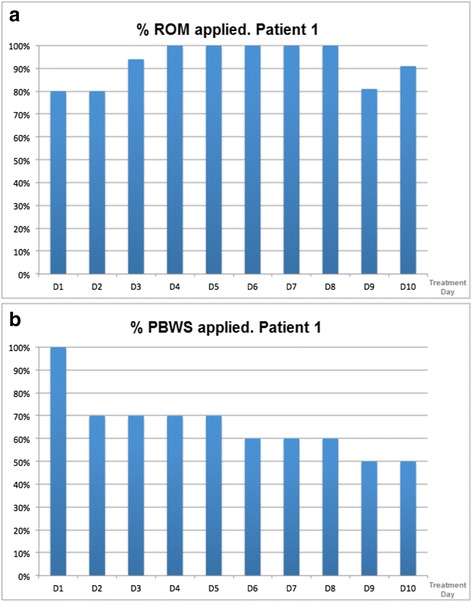
Evolution of therapy parameters in Patient 1. **a** Percent of the range of motion of a healthy user programmed in the robot. **b** Percent of the subject body weight supported by the platform. These parameters were adjusted during the treatment in order to tailor the therapy to each patient

With the aim of accommodating the patients to the robotic platform and following therapists’ recommendations, all of them were completely suspended during the first sessions (PBWS of 100 %), and the PBWS was gradually decreased along the course of 5 weeks of the study (Fig. 2). This approach allowed the subjects to gradually get used to support their weight on their own legs. In the same way, the percent of ROM applied for each joint respect to a normal gait pattern (Fig. 2) and the gait speed were updated during the therapy with the purpose of increasing the difficulty of the exercise.

According to the pre-measured capabilities for each child and following recommendations from our clinical partners, the parameters were selected before to start the robotic therapy (Table 2).

**Table 2:** Selected parameters according to patients’ capabilities

	% ROM		% PBWS		Gait speed (m/s)	
	Beginning	Desired End	Beginning	Desired End	Beginning	Desired End
Patient 1	80	100	100	50	0.172	0.210
Patient 2	85	90	100	60	0.159	0.210
Patient 3	73	88	100	65	0.133	0.188

The designed therapies were based on two key features: an interface to correct the user’s posture during robot-assisted walking and a selective impedance control to achieve the tailored therapy related to the patient’s needs.

### Postural control therapy

**Fig. 3:**
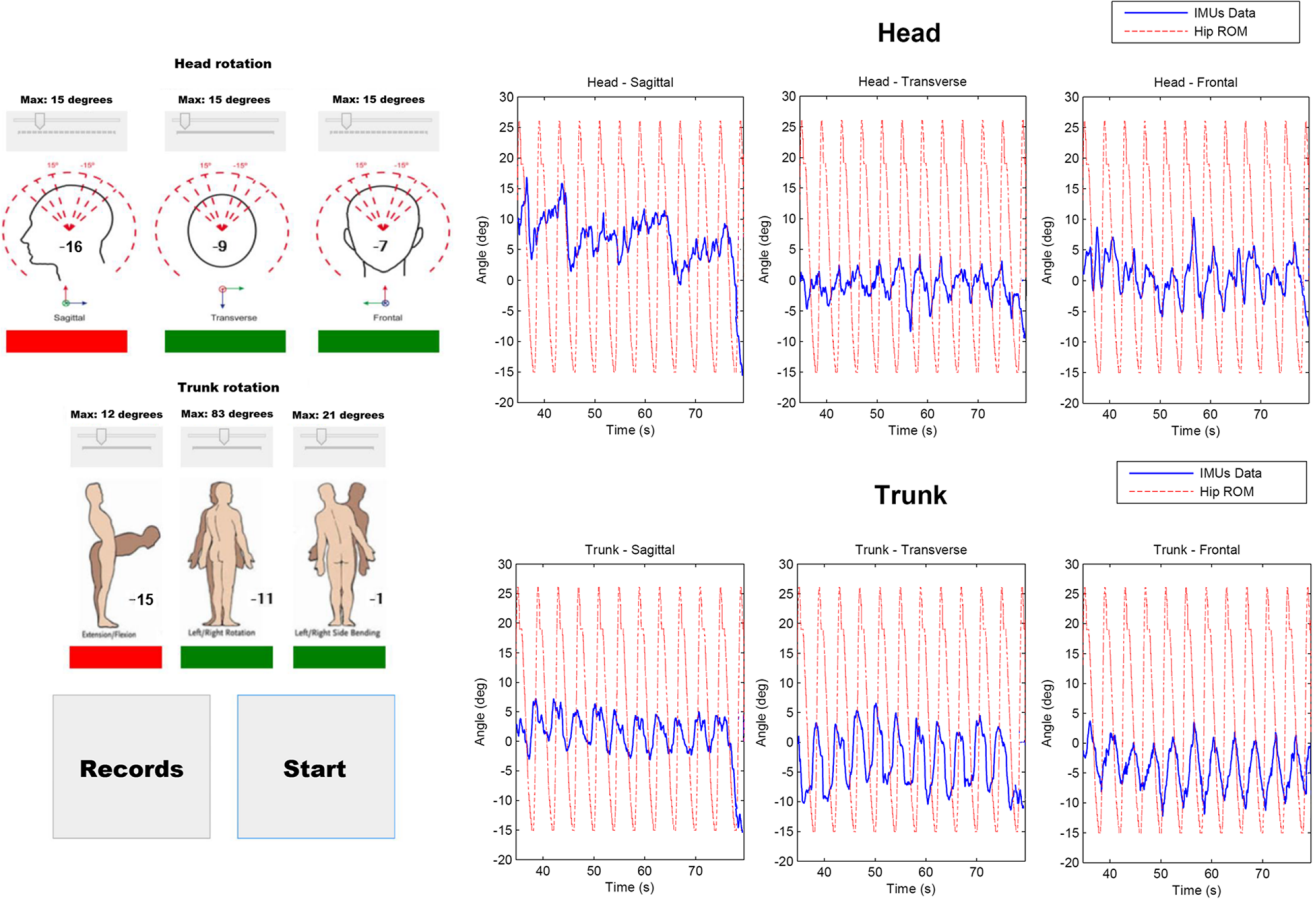
IMUs based interface to give biofeedback of postural control in head and trunk. The graphics show IMUs data collected in real time for head and trunk in three planes (*blue lines*), and these are compared with hip ROM (*red lines*). The *red* squares represent postures out of the limit values (acoustic feedback playing)

The biofeedback strategy for postural control was based on inertial measurement unit (IMU) sensors (Fig. 3). Two IMU sensors, placed on the user’s chest and head, measured the orientation of the trunk and head respectively. The procedure based on this approach consisted in giving acoustic feedback to the users when their trunk or head were not in a proper position, which was defined by the clinicians (see clinical interface on the left of Fig. 3). The measures provided by this biofeedback strategy were important during the execution of the therapy: while the exoskeleton corrected the patients’ gait, the postural control strategy motivated them to maintain a proper posture during ambulation [6, 7, 11]. Figure 3-right shows an example of recording data in real time from IMUs-based system, where the measured angles for head and trunk (blue lines) in three spatial planes are compared to the ROM of the left hip during walking (red lines). Red squares in Fig. 3-left represent posture out of the permitted range and where acoustic feedback was given.

### Assist as needed therapy

The AAN philosophy was included into the control via a selective impedance control strategy [12], which was implemented in specific and selected subtasks during walking therapy. This approach increased the patient’s active participation during the exercise performance.

The method considers the interaction torque between the user and the exoskeleton, allowing a variable deviation from the predefined reference trajectories. As a result, CPWalker attempts to prevent undesired efforts on lower limbs and to take advantage of patient’s residual movements.

## Results

Children’s 3D gait assessments without the aid of CPWalker were conducted before and after the robot-based therapy. This analysis provided kinematic data and temporal-spatial parameters that were used to evaluate the progression of the therapy.

**Fig. 4:**
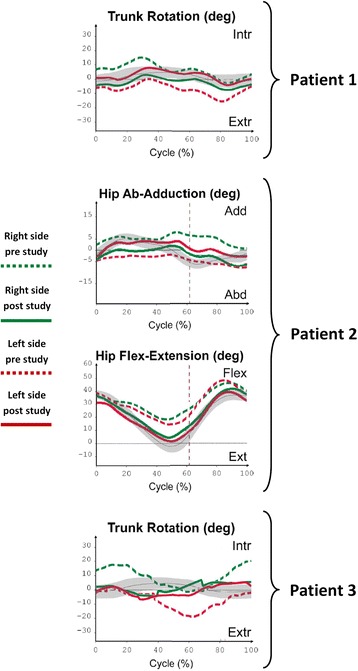
Outcomes from kinematic analysis in Patient 1, 2 and 3 without the robotic aid. The graphics show the improvements for each patient depend on the focus of each therapy. *Green* lines are referred to right side and *Red* lines to left side. Dashed lines correspond to 3D studies done before the robotic treatment and Continuous lines to 3D studies done after 5 weeks of robot-based therapy

After 5 weeks of robot-based training with CPWalker system the three children improved the mean velocity, cadence and step length with each leg (Table 3). Additionally, taking into account the kinematic analysis, the three subjects progressed in their gait as Fig. 4 and Table 3 shown. Post-3D studies revealed that the trajectories for right and left lower limbs are closer to the normal values when compared to pre-3D studies. All children succeeded the goals proposed on Table 2.

**Table 3:** Comparison between Pre and Post studies

		Pre 3D analysis		Post 3D analysis		Normality
Patient	Parameter	Right side	Left side	Right side	Left side	
Patient 1	Mean velocity (m/s)	0.40 ± 0		0.49 ± 0		1.20 ± .20
	Cadence (step/min)	73.80 ± 6.00		75.80 ± 7.97		129.60 ± 8.40
	Step length (m)	0.30 ± .01	0.24 ± .04	0.33 ± .02	0.27 ± .01	0.58 ± .06
Trunk rotation	Percentage range (max-min)	286.29 %	248.71 %	204.84 %	233.87 %	100 %
	Maximum peak	14.83°	0°	4°	10°	4.80°
	Minimum peak	−2.92°	−15.42°	−8.70°	−4.50°	−2.40°
Patient 2	Mean velocity (m/s)	0.60 ± .10		0.80 ± 0		1.20 ± .20
	Cadence (step/min)	102.20 ± 12.65		120.8 ± 9.38		129.60 ± 8.40
	Step length (m)	0.31 ± .03	0.24 ± .05	0.40 ± .01	0.38 ± .02	0.58 ± .06
Hip ROM flex-ext	Percentage range (max-min)	70.63 %	85.82 %	95.19 %	96.20 %	100 %
	Maximum peak (flexion)	46.40°	48.40°	41.90°	39.50°	37.10°
	Minimum peak (extension)	18.50°	14.50°	4.30°	1.50°	−2.40°
Patient 3	Mean velocity (m/s)	0.20 ± 0		0.40 ± 0		1.20 ± .20
	Cadence (step/min)	45 ± 3.70		75 ± .60		129.60 ± 8.40
	Step length (m)	0.23 ± .05	0.30 ± .05	0.32 ± .02	0.33 ± .04	0.58 ± .06
Trunk rotation	Percentage range (max-min)	343.55 %	350.48 %	224.52 %	234.35 %	100 %
	Maximum peak	20°	2.60°	8.25°	6.28°	4.80°
	Minimum peak	−1.30°	−19.13°	−5.67°	−8.25°	−2.40°

Temporal, spatial parameters and kinematic data related with the selected improvements for each patient (correspondence with Fig. 4)

As the therapies were individually tailored for each patient, the results have to be understood as separate case studies.

## Discussion and conclusion

This contribution has presented the importance of considering novel robot-based strategies for gait rehabilitation and its previous results from a validation using the CPWalker device with pediatric population. The strength of the robotic platform used in this study is the capability of AAN gait training with displacement in real environment while the apparatus provides feedback in order to correct the child’s posture. These functions enabled the definition of tailored therapies for each patient.

Although outcomes are quite promising, we acknowledge that the population size of this pilot trial, the reduced time of intervention (only 10 sessions) and the short-term follow up are the main limitations of this research. Future work will be focused on performing a proper clinical evaluation with an increased number of patients. These studies are necessary to determine if gains will have long-term and lasting impact for children with CP and other similar disorders.

Additionally, we are currently evaluating the effectiveness of a non-invasive way for promoting a more active participation of central nervous system into the rehabilitation strategy in order to allow the implementation of a “Top-Down” approach [13].

## Additional file

Additional file 1: Video illustrating the rehabilitation process based on the CPWalker robotic platform. (MP4 17260 kb)

## Abbreviations

AAN: Assist as needed
AFO: Ankle-foot orthoses
CP: Cerebral palsy
DOF: Degrees of freedom
GMFCS: Gross motor function classification system
HSP: Hereditary spastic paraparesis
IMUs: Inertial measurement unit
PBWS: Partial body weight support
ROM: Range of motion
